# Dental Health Status and Hygiene in Children and Adolescents with Type 1 Diabetes Mellitus

**Published:** 2016-07-30

**Authors:** Rezvan Rafatjou, Zahra Razavi, Soudeh Tayebi, Maryam Khalili, Maryam Farhadian

**Affiliations:** ^a^ Department of Pediatric Dentistry, School of Dentistry, Hamadan university of Medical Sciences, Hamadan, Iran; ^b^ Department of Pediatrics, School of Medicine, Hamadan University of Medical Sciences, Hamadan, Iran; ^c^ Modeling of Non-communicable Disease Research Center, Department of Biostatistics, Hamadan University of Medical Sciences, Hamadan, Iran

**Keywords:** Hemoglobin A1c Protein, Child Health, Oral Hygiene, Periodontal Disease, Type 1 Diabetes Mellitus

## Abstract

**Background:** There is disagreement on the effect of diabetes on oral hygiene. The purpose of this study was to assess the oral health and hygiene status of type 1 diabetic patients.

**Methods:** In this case control study, periodontal health and hygiene of 80 children and adolescents (5–18 yr of age) with type 1 diabetes mellitus referred to Pediatric Endocrine Clinic of Besat Hospital Hamadan Iran 2013 – 2014 and 80 non diabetic control subjects were clinically assessed. The required data such as sex, age, duration of the diabetes, type and number of insulin injections per day were obtained from self-administered questionnaire and the patient’s medical records. Participants in both groups were examined for Decay-missing- filled teeth (DMFT); dmft (for primary teeth), oral hygiene using O'Leary plaque index (PI) and gingivitis index (GI). *P*<0.05 was considered significant.

**Results:** The mean age of the study and the control group was 12.5±4.05 and 12.08±3.47 yr, respectively. There were no significant difference between two groups in terms of DMFT (*P*=0.158) and PI indices (*P*=0.373). The GI index difference was statistically significant in diabetic group (*P*=0.001). Interestingly, a higher dmft index was observed in the control group (*P*=0.008). In diabetic groups, GI and DMFT index increased significantly with duration of diabetes.

**Conclusions:** Apart from higher scores of GI index, frequency of oral and periodontal disease was not different in diabetic patients compared with healthy subjects. Findings of present study are insufficient to support a significant effect of diabetes on increasing the risk of oral and periodontal diseases. However, diabetic children and adolescents should receive oral hygiene instructions.

## Introduction

Type 1 diabetes mellitus (T1DM) is a common metabolic disease of childhood. About 1 in every 400-600 children and adolescents has T1DM. In adults, T1DM constitutes approximately 5% of all diagnosed cases of diabetes chronic illness^1,2^. A 2011 report from the US Centers for Disease Control and Prevention (CDC) estimated that approximately one million Americans have T1DM^1^. Onset most often occurs in childhood, but the disease can also develop in adults in their late 30s and early^1^.

In this type of diabetes, an autoimmune destruction of the beta cells of the pancreatic islets leading to defects in insulin secretion. This results in persistent hyperglycemia and the clinical manifestation of the disease with dependence on exogenous insulin to prevent ketosis. The disease manifests itself in genetically predisposed individuals (polygenic genetic predisposition). Oral disease include xerostomia, periodontal disease (gingivitis and periodontitis), dental abscesses, tooth loss, soft tissue lesions, dry mouth and dental caries have been proposed as the 6^th^ most prevalent complication of diabetes mellitus following the other diabetic complications^3-5^. The co-morbid presence of various inflammatory diseases and soft tissue pathologies in oral cavities in turn, adversely affect glycemic control and the treatment of oral complications can lead to improved metabolic control in diabetes patients^6-8^. Although patients with diabetes face a significantly higher risk for oral complications than healthy subjects^9-11^, there is controversy on the impact of diabetes on oral and periodontal diseases and the mechanisms through which this occurs^12,13^.

Considering the fact that some studies have reported a high prevalence of diabetes in Iran^14^ and controversies about the impact of diabetes on oral health’s status of T1DM and lack of public awareness in this regard further studies in this area is reasonable. Accordingly, we aimed to evaluate the oral health status of young patients with T1DM compared to healthy subjects in Hamadan west province of Iran.

## Methods

In this case control study, oral health and hygiene of children and adolescents (5–18 yr of age) T1DM were clinically assessed.

The protocol was performed according to the principles of the declaration of Helsinki. The study protocol was approved by the Ethical and Research Committee of Hamadan University of Medical Sciences in 2013. Because all participants were under eighteen years old, written informed consent was obtained from all of their parents.

The study group consisted of 80 subjects (5-18 yr, 46 females, 34 males) diagnosed with T1DM who were followed in Pediatric Endocrine Clinic of Besat Hospital Hamadan Iran 2013 – 2014 and of 80 healthy subjects (5-18 yr,46 females, 34 males) randomly recruited from the school population. Both groups were matched in terms of age, gender, education and socioeconomic status. The number of males and females were equal in both groups and in order to assess the age consistency between two groups independent sample *t*-test was used. The sample size was calculated based on variance reported by previous related studies. Moreover, the sample size in the present study was comparable or even higher than many previous studies conducted on T1DM patients^15-17^.

T1DM was defined based on the EURODIAB criteria^18,19^. Clinical periodontal evaluations were performed by one pediatric dentist and fast blood sugar test was conducted to prove the health condition of healthy people.

The required information related to the diabetic patients including duration of diabetes, age at diagnosis, insulin regimen (twice daily, multiple daily insulin injections or continuous subcutaneous insulin infusion), mean of HbA1c within 1 years of enrollment profiles was collected from medical records. The degree of glycemic control evaluated by measuring HbA1c levels as: Good (HBA1c≤7), fair (HBA1c= 7-8) and poor (HBA1c >8)^20^. Measurements of glycated hemoglobin were performed by D-10 high-performance liquid chromatography.

Inclusion criteria:‏ All Type 1 DM patients‏ with diabetes duration of more than 1 yr who received at least two daily insulin‏ injections‏.

Exclusion criteria: Type1 diabetic patients with diabetes duration of less than 1 yr.‏ Those without regular follow-ups, or with other chronic diseases and on medications‏ that could influence the oral health status (as antibiotics and antiepileptic) were excluded.‏ Those without the required information of the study were also excluded.

Patient information such as age, sex, parental education, level living location (rural or urban) and parents' job and oral and dental information such as the number of brushing per day, the use of dental floss and mouth wash were gathered through the questionnaire. The required information related to the diabetic patients including duration of diabetes, age at diagnosis, Insulin regimen (twice daily, multiple daily insulin injections or continuous subcutaneous insulin infusion), mean of HbA1c within 1 yr of enrollment (for to obtain a measure of diabetic control), mean fasting and 2 hours post prandial blood glucose within 1 years of enrollment was collected from medical records. O'Leary plaque index (PI) was employed for assessing the oral hygiene condition of patients. In order to measuring the PI index^4^, the detective tablet was used to evaluate the color deposits (microbial plaque) on four tooth surfaces at the dent gingival junction. The index is calculated by dividing the number of surfaces with a plaque on the total number of surfaces^21^. DMFT were another index used in this respect^22,23^. In the present study, the term DMFT is related to permanent teeth, while the term dmft was adopted to express the index in primary teeth. The severity of gingivitis (GI) was evaluated using a periodontal probe and divided in three groups; furthermore, gingivitis index (GI) was used for determining the severity of gingivitis. The index has a range from zero to three. For calculating the index, all four-tooth surfaces except the occlusal one were assessed and then a score within 0-3 were assigned to each tooth surface. The process was completed for all teeth, and then the summation of scores was divided on the total number teeth surfaces (teeth number × 4). According to the final score obtained in the previous step, the severity of gingivitis was categorized as follows; score 0.1-1 the mild inflammation, score 1.1-2: moderate inflammation, score >1.2-2 (11): severe inflammation. In the present study, several periodontal indices were also assessed^24^.

Bleeding on probing, known as BOP index, was the first periodontal index investigated by the present study to assess periodontal status of subjects. In order to assess the oral health of participants in term of this index, the region where gingiva and teeth come to contact to each other is gently stimulated by a periodontal probe. Bleeding after stimulation is indicative of inflammation or erosion in gingival sulcus^25,26^.

Probing depth, "defined as the distance between the gingival margin and the bottom end of the periodontal pocket" ^27^ was another periodontal index investigated measured by a periodontal probe so that the penetration depth of the probe was regarded as the depth of periodontal pocket^28,29^.

Furthermore, clinical attachment loss was another index assessed by the present study. This index is the length between the cement enamel junction (CEJ) and the bottom end of periodontal pocket ^28,29^. According to this definition, the value of clinical attachment loss should be equal or higher than probing depth.

### Statistical analysis

The data obtained from each group were compared. Analyses were performed using Independent *t*-test by SPSS 16 (Chicago, IL, USA) software package. Moreover, for evaluating the correlation between variables, based on the type of variables, Spearman and Pearson's correlation coefficients were employed. *P* values less than 0.05 were considered statistically significant.

## Results

Overall, 160 children (80 cases suffering from T1DM and 80 healthy subjects) were investigated. As two groups were matched in terms of gender composition, each of them was composed of 34 males and 46 females. Moreover, the average age of the case group was 12.5 yr with a standard deviation of 4.05 yr, and the average age of the control group was equal to 12.09 yr with a standard deviation of 3.47 yr. In order to check the consistency of age between two groups, independent sample *t*-test was employed. There was no significant age difference between two groups (*P*=0.491).

Baseline dental and periodontal characteristics of the study population are summarized in Table 1. GI index value was found significantly higher in diabetic group (*P*=0.001) in comparison with the control group. Whereas, compared with the diabetic group dmft index was significantly higher in control group (*P*=0.008). There was no significant difference between the number of permanent decayed teeth of the two groups (*P*=0.157), however the number was higher in the case group. Moreover, the independent *t*-test revealed that the number of primary decayed teeth was significantly higher in the control group (*P*=0.011).

**Table 1:** Oral hygiene indices for the case and control groups

**Parameters**	**Diabetics**				**Non-diabetics**				***P*** ** value**
	**n**	**Min**	**Max**	**Mean (SD)**	**n**	**min**	**max**	**Mean (SD)**	
DMFT	73	0.00	12.00	3.78 (3.24)	75	0.00	12.00	3.08 (2.74)	0.158
DMFT	28	0.00	11.00	2.52 (3.29)	33	0.00	12.00	5.36 (3.21)	0.008
GI	80	0.00	1.92	0.45 (0.49)	80	0.00	1.12	0.26 (0.24)	0.001
PI	80	8.93	95.60	21.38 (43.63)	80	11.20	87.50	46.57 (20.11)	0.373

Although compared with the diabetic group the number of visits to the dentist per years was significantly higher in the control group brushing per day, the use of dental floss and mouthwash were similar between two groups. Analysis of the data also demonstrated no statistically significant differences in attachment loss, probing depths, recession, plaque index, and bleeding on probing between two groups. However, comparisons based on site-specific measurements showed that the gingival index to be somewhat higher among the diabetics group (*P*=0.002).

Although the periodontal disease indexes increased with getting worse of diabetes control (increased Hb1C) but‏ apart from GI no significant difference was found with other indexes. We investigated the association between periodontal disease and diabetes-related variables. The association between duration of diabetes and GI and DMFT was statistically significant (*P*=0.002, and *P*=0.00 respectively). There was also a positive and statistically significant association between GI and mean fasting blood glucose and mean Hb1C (*P*=0.001, *P*=0.006, respectively). With the exception of DMFT, there was no statistically significant association between gender of diabetic patients and oral periodontal disease. No significant difference was found between periodontal disease, type, and number of insulin injections. The results of diabetes-related variables for the case group are given in Table 2.

**Table 2:** Characteristics of control group obtained from hospital records

**Variables**	**Number**	**Min**	**Max**	**Mean (SD)**
FBS (mg/dl)	80	83.50	324.00	170.98 (50.77)
2hpp (mg/dl)	80	68.50	475.00	225.54 (88.93)
HbA1c (%)	80	5.80	13.20	8.54 (1.62)
Patients' age (yr)	80	1.00	14.00	7.03 (3.28)
Duration of diabetes (yr)	80	2.00	17.00	5.46 (3.48)

## Discussion

Periodontal disease is a major complication of diabetes mellitus and treating periodontal conditions results in improved metabolic control. On the other hand, importance of oral health and its impact on glycemic control is unknown for many patients and practitioners^21^.

The objective of this study was to describe the associations between oral health variables and T1DM. In current study apart from higher scores of GI index, frequency of oral and periodontal disease were not different in children and adolescents with T1DM compared with healthy subjects which is contrary to the results of some previous investigators^4,6,30,31^. For example Orbak et al.^4^ illustrated that diabetes mellitus plays an important role in patients' oral health status and the need for treatment. Popławska-Kita et al.^31^ revealed T1DM increases the risk of periodontal disease‏ .In their study periodontitis was found in 57.9% of diabetic patients (even with good metabolic control) compared with 15.0% of healthy subjects.

In our study, GI index was found significantly higher in diabetic group compared to healthy control subjects. Siudikiene et al.^32^found a higher prevalence of gingivitis in young patients with T1DM in Lithuania (27% versus 13%). Similarly, young cases with T1DM had significantly increased severity of inflammatory gingival disease compared with age-matched control group^33^. Bissong et al.^34^, observed a larger number of gingivitis (23.5%); periodontitis (24.8%) dental caries (19.5%) and oral candidiasis (21.5%) in 149 diabetic population in‏ comparison healthy subjects.

Our findings also showed that subjects with poor glycemic control as evident by the higher HbA1c had greater gingival inflammation, similar to previous stusdies^20,30^.It can be assumed that sustained high blood sugar levels over time appears to increase destruction of periodontal tissues as a result of microvascular effects of advanced glycosylation end products and chronic inflammatory mediator secretion or abnormally high degree of inflammation^7^. Uncontrolled diabetics may decrease salivation and change in the composition of saliva^35^. Hyposalivation may be involved in the pathogenesis of periodontal disease. Contrary to our study, Pinson et al^33^ and Busato et al^36^ did not find a positive correlation between the glycemic control and studied oral hygiene.

In this study, a positive correlation between duration of diabetes and missed/decayed/filled teeth and the severity of gingivitis was found. This finding is reasonable because, like other complications of diabetes, the risk of oral and periodontal disease tends to increases over time.

We did not notice differences between the numbers of missing permanent teeth in both examined groups. However, the higher number of this parameter in diabetic group should not be overlooked.

Interestingly, a higher dmft index was observed in the control group. This means that the incidence of missed, decayed, or filled primary teeth is high in healthy subjects. Educational efforts must be reinforced mainly in children and adolescents, emphasizing the importance of oral and periodontal health. Therefore, health care providers should pay more attention to this area.

Analysis of maximum values of periodontal disease index reveals higher level in diabetic girls than in female controls. We do not have an explanation for this difference‏.

This study indicates that frequency of dental examination in diabetic patients is lower than general population, in agreement with previous studies^37,38^, emphasizing the lack of awareness of‏ young diabetic patients about this important health issue. We assume that diabetic patients and their family are often involved in management and treatment of blood glucose‏ and hence, other aspects of general health including oral hygiene oral health is under consideration .Therefore, there is need to increase the general information of young diabetic patients in this respect.

Results of this study are limited by the small sample size and short diabetes duration. Further studies with larger sample size and longer follow up periods involving the oral health status of young T1DM may reveal different results. These studies need to evaluate the prevalence and progression of oral disease and to assess impact of periodontal therapy on improvement of metabolic control of young T1DM.

## Conclusions

Although the evidence of current work suggests that diabetes was a risk factor for high frequency of gingivitis in young diabetic patients, the results of this study is insufficient to support a significant impact of diabetes on increasing the risk of oral and periodontal diseases. We suppose that host factors could modulate metabolic influence of diabetes. However, periodic assessment of oral health status of patients should be promoted as integral components of diabetes management and the dentist should be a part of the multidisciplinary team that assists individuals with T1DM. In addition, diabetic patients should receive oral hygiene instructions.

## Acknowledgments

This paper was extracted from a PhD thesis by Maryam Khalili. The authors would like to express their thanks to all of the participants and their parents for their cooperation and completion of this work.

## Conflict of interest statement

The authors declare that they have no conflict of interests associated with this study.

## Highlights

- Diabetes mellitus is a risk factor for gingivitis. Other oral and periodontal disease
- In diabetic patients is not higher than the healthy subjects .
- There is a relationship between duration of diabetes and missed/decayed/filled teeth and the severity of gingivitis .
- Diabetic patients require periodic dental examinations. They should also receive oral hygiene instructions.
